# Evaluation report on the causal association between humidifier disinfectants and lung injury

**DOI:** 10.4178/epih.e2016037

**Published:** 2016-08-18

**Authors:** Mina Ha, Soon Young Lee, Seung-sik Hwang, Hyesook Park, Seungsoo Sheen, Hae Kwan Cheong, Bo Youl Choi

**Affiliations:** 1 Department of Preventive Medicine, Dankook University College of Medicine, Cheonan, Korea; 2 Department of Preventive Medicine & Public Health, Ajou University School of Medicine, Suwon, Korea; 3 Department of Social and Preventive Medicine, Inha University School of Medicine, Incheon, Korea; 4 Department of Preventive Medicine, Ewha Womans University School of Medicine, Seoul, Korea; 5 Department of Pulmonology, Ajou University School of Medicine, Suwon, Korea; 6 Department of Social and Preventive Medicine, Sungkyunkwan University School of Medicine, Suwon, Korea; 7 Department of Preventive Medicine, Hanyang University College of Medicine, Seoul, Korea; 8 Academic Committee, Korean Society of Epidemiology

**Keywords:** Causality, Humidifiers, Disinfectants, Hill’s criteria, Lung injury, Association

## Abstract

**OBJECTIVES:**

As of November 2011, the Korean government recalled and banned humidifier disinfectants (HDs) from the market, because four case-control studies and one retrospective epidemiological study proved the association between HDs and lung injury of unknown cause. The report reviewed the causal role of HDs in lung injury based on scientific evidences.

**METHODS:**

A careful examination on the association between the HDs and lung injury was based on the criteria of causality inference by Hill and the US Surgeon General Expert Committee.

**RESULTS:**

We found that all the evidences on the causality fulfilled the criteria (strength of association, consistency, specificity, temporality, biologic gradient, plausibility, coherence, experiment, analogy, consideration of alternative explanations, and cessation of exposure), which proved the unknown cause lung injury reported in 2011 was caused by the HDs. In particular, there was no single reported case of lung injury since the ban in selling HDs in November 2011 as well as before the HDs were sold in markets.

**CONCLUSIONS:**

Although only a few epidemiological studies in Korea have evaluated the association between lung injury and the use of HDs, those studies contributed to proving the strong association between the use of the HDs and lung injury, based on scientific evidence.

## INTRODUCTION

The incident by humidifier disinfectants (HDs) in 2011 was a large-scale and unprecedented environmental catastrophic case. Since a notification by the infection control department at a university hospital in Seoul in April 2011 resulted in the epidemiological investigation by Korea Centers for Disease Control and Prevention (KCDC), studies on HD-induced lung injury have been performed. Evidence on the cause of these cases begun to be inferred from these study results. Based on the initial hospital-based case-control study and the preliminary result of inhalation toxicity test in mice, the government withdrew HDs from the market and banned its sale in order to prevent additional damages in November 2011. No similar incidence of lung injured patients was observed afterwards [1].

However, despite the ban on the sale of HDs, there has been no progress in the treatment and compensation for victims affected by their use and it was failed even to grasp the problem magnitude, i.e., the number of victims. A lawsuit brought by some victims against the company and the government was dismissed. Fundamental investigation by the prosecution began in early 2016, and in the process, unethical behaviors by relevant companies and systemic problems have been discovered. Lately, with the establishment of a special investigation committee on HDs by the National Assembly, various systemic and political measures were being considered with respect to compensation for victims including recognition of the impact of the disease on other organs, as well as other respiratory diseases. Moreover, a legal resolution on the prosecution’s indictment, including the possibility of a causal role for HDs in health issues, was yet to be reached in court.

Based on this situation, this report reviewed the causal role of HDs in lung injury based on scientific evidences, and discussed issues to be considered in establishing a possible causal association with other diseases other than lung injury.

## MATERIALS AND METHODS

The counterfactual concept is often applied to prove that certain exposure can cause a specific disease in a population. It is assumed that whether a certain factor is the cause of the disease can be decided by comparing the prevalence of the disease under exposure (fact) with the prevalence of the disease without exposure (counter-fact) of the same subjects (individual or population) in the same environment [2]. However, in reality, although observing the fact is possible, observing the counter-fact in the same situation may not be possible. Instead, a method to compare the prevalence of the disease in others but similar exposure and environmental conditions is adopted in subjects with similar characteristics. This method generates the association between exposure and the disease. Therefore, this is different from causality, which can be obtained by comparing the prevalence of the disease in the fact and counter-fact. In other words, because of the similar but not identical conditions such as the subject and the environment, confounding and mediating factors develop with variables of such difference. Therefore, logical inference from various perspectives is required to interpret the association obtained from the result of an epidemiological observation like causality. In other words, once it is verified that the association observed in an epidemiological study is not from various biases and by chance (statistical significance), then the criteria of causality are met and this determination should be decided [3].

Although recent models have been suggested, particularly to explain chronic disease related causality, this report was based on the criteria of causality inference by Hill [4] and the US Surgeon General Expert Committee [5], which are still broadly used as practical criteria for causal inference by epidemiologists and health policy experts.

## RESULTS

### Epidemiological reports of the association between humidifier disinfectants and lung injury

As of 2016, a total of six case series reports detailing the clinical, radiological and pathological patterns of lung injury and its prognosis have been published [6-11]. Five epidemiological studies generating estimates on the association of lung injury with HDs have been published (Table 1) [12-16].

There were four case-control studies: an epidemiologic study initially performed immediately after a report of lung injury from a university hospital to the KCDC in 2011 [12]; a case-control study matching sex, age, and date of diagnosis in three controls in children [13]; a case-control study matching sex, age, residential area, and experience of childbirth in a community control group [14]; and a study comparing patients exposed to humidifiers more closely and longer to other family members, as well as to a control group within a family [15]. In order to exclude bias due to confounding variables in this study, either matching for major confounders or statistical adjustment in analysis was conducted. Particularly, in the initially performed epidemiological study [12] as well as a community-based case-control study [13], it could be inferred that the possibility of recall bias was minimized, because at the time, neither the patients nor the investigators recognized that HDs was a possible cause. Excluding the study using a family control group, the other three case-control studies reported as few as 16 to 18 patients, but since 90% to 100% of the patient groups used HDs, the possibility of statistical chance could be excluded. A retrospective cohort study that recruited patients nationwide showed that of all HD victims, female and children were at a greater risk, and the risk increased in proportion to the dose of exposure [16].

### Evaluation of evidence for causality according to the causality criteria

Based on the results of epidemiological, clinical, toxicological and experimental studies, the causality of lung injury by HDs was summarized by applying Hill’s nine criteria and the US Surgeon General Expert Committee’s nine criteria as follows (Table 2).

First is the strength of association. According to the result of the above epidemiological study, the risk estimate between HD and lung injury was as low as 2.7 times, and as high as 116.0 times, showing a significantly strong association [12-14].

Second is consistency. Although each epidemiological study had different populations (adults [12-14] vs. children [13]), the study design (case-control [12-15] vs. retrospective cohort [16]) or other control groups (hospital [12,13], community [14], and family [15]) showed consistency.

Third is specificity. This lung injury could not be explained previously by other common types of lung diseases such as cases of infectious lung disease, those caused by other viruses or bacteria, or immunologic lung diseases, by clinical and pathological findings [7-9]. And it could not be explained by any other environmental factors than HDs, as described in the epidemiological studies [12,13]. From this perspective, it was recognized that there was a specific association between the exposure to HDs and this form of lung injury.

Fourth is temporality. Considering that these cases of lung injury had not been reported before HDs were on the market, and there have been no additional case reports since the ban of its sale in November 2011, temporality has been met.

Fifth is the dose-response relationship. Three epidemiological studies have shown consistent results, that the risk of incidence, as well as mortality increased with larger doses, longer durations [13,15], and closer proximity [14] of exposure to HDs.

Sixth is plausibility. The most common size of aerosol sprayed through humidifier was 30 nm to 50 nm. Particles of this size were found to have reached the bronchioles and precipitated [1], suggesting that the chemical component of HDs can reach the bronchioles through aerosols. Moreover, when the diluted HD in the concentration used in the market was dropped on the bronchus of an animal, lung injury was induced, suggesting that the concentration of diluted HD marketed was sufficient to induce toxicity. These results showed that it was plausible for sprayed diluted HDs into the air to reach the bronchioles through inhalation, and induce lung injury.

Seventh is consistency with previous knowledge. Previously known lung lesions due to inhalation toxicity have characteristics of lobular, diffuse infiltration and peri-bronchial infiltration that are peculiar to lung injury [7,9]. As it was not considered that diluted disinfectants would be used for humidifiers in Australia or the US, inhalation toxicity data was not available. However, although there was no inhalation toxicity evaluation data on the major component of HDs because of low volatility at room temperature, various toxic evidences from oral or dermatological contact have been reported [1]. These facts show that the association between HDs and lung injury is consistent with previous knowledge.

Eighth is experimental evidence. In a cellular toxicity test exposing normal pulmonary cells to the major components of HDs, dose-response toxicity was found. The result of the evaluation of reactive oxygen production also showed a dose-response relationship [1]. In an inhalation animal experiment of diluted HD at the concentration sold in the market, it was verified that the same histopathological findings found in the lungs of the patients with lung injury were reproducible [17].

Ninth is analogy. Ardystil syndrome, an interstitial lung disease developed in workers using spray paint in the western country in the 1990s, was similar to the lung disease caused by HDs [18]. This was relevant because acramin, a component of spray paint, has a very similar chemical structure to polyhexamethylene guanidine found in HDs.

Unlike Hill’s criteria, the US Surgeon General Expert Committee suggested to consider alternative explanations as a causality criteria. First of all, the possibility of fungi as a cause of lung injury. Although there were no other significant risk factors than HDs in the studies of lung injury, the first epidemiological study showed significant association between fungi at room reported by questionnaire administration with lung injury, even after adjustment for various variables [12]. Association with fungi was substantially small compared to association with the HDs; in addition, there was no consistency of results, as there was no significance in the analysis of different control groups. Furthermore, fungi were not detected in the patient’s specimen. Above all, there was no significant association between fungi and lung injury among the study in children and community-based case control study [12,13]. Thus, the possibility that fungi may be a direct cause of lung injury is very low, and it is reasonable to infer that fungal proliferation has been induced by prolonged use of humidifier itself. Secondly, the possibility that humidifier fever or white-dust related fever, which has been reported during humidifier use, could be responsible for lung injury. Humidifier fever or hypersensitivity pneumonitis is caused by toxic substances (endotoxins) from colonized bacteria in the humidifier [19], and white-dust related fever is a lung disease caused by inhalation of metal deposits such as calcium and magnesium present in the water of humidifier [20]. As these diseases have distinctive and well-described features in clinical, radiological, and pathological findings when compared to HD-induced lung injury, it was difficult to see either of them as the cause of lung injury.

In addition, the US Surgeon General Expert Committee suggested that disease risk should decrease after cessation of exposure if it was a cause. The fact that there have been no new cases of this form of lung injury since HDs have been withdrawn and banned from the market in November 2011 meets the criteria of decrease in risk upon cessation of exposure.

## DISCUSSION

In summary, the unusual association between the HDs and lung injury met all nine Hill’s criteria and the nine criteria of US Surgeon General Expert Committee. Therefore, it could be inferred that there was a very strong scientific evidence of HDs as the cause of lung injury. However, the inhalation toxicity of HDs might not be limited to lung injury. As indicated in previously published reports, lung injury is considered as a disease developing in a high-risk group with a very high exposure level. There was a possibility that mild diseases could have developed in people exposed to a lower level. Moreover, damage of other organs than the lung was suspected. In fact, rhinitis or asthma was often reported in victims, as well as symptoms in other organs [16]. Moreover, abnormal cardiovascular findings and liver injury were found in a fish toxicity test [21]. It could not be inferred that HD was associated only with lung injury, and the specificity of the association of lung injury could be refuted. However, although exposure to numerous HDs was associated with a specific form of lung injury, like smoking which is a cause of lung cancer, there was the possibility that exposure can lead to non-specific symptoms such as worsening of previous lung diseases, asthma or rhinitis when the exposure level of HD was relatively low. In fact, specificity may not be a necessary condition in the review of causality [5].

On the other hand, most environmental diseases could be developed from multiple causes. It is more reasonable to infer various factors as a necessary cause or a sufficient cause working simultaneously and complexly. Also, environmental diseases develop under the influence of the complex and multi-dimensional interactions; from biochemical reactions at a microscopic level, individual behavioral factors and to the microscopic level such as the physical, chemical, social, and psychological environment [5]. It was difficult to exclude the involvement of HDs in the incidence of lung injury to such an extent that it would be attributed to interaction with other factors. Interaction with personal sensitivity and economic as well as social capacity determines the coping capacity. In other words, although exposure to HDs was a necessary factor for the incidence of lung injury and it was certainly a direct cause, other possible factors could co-exist in the development of lung injury as a cause of disease development. Identifying the causality between a certain factor and a specific disease incidence was very fundamental for disease prevention and treatment, but it was not easy to conclude causality considering non-specificity, possible co-existence of various factors, and possible interaction of various factors in most diseases. Although the use of HDs in 2011 was limited to Korea and there were only a few epidemiological studies, they were significant in their contribution to rapid and scientific identification of the cause of this disease.

In conclusion, after reviewing the causal role of the HDs in lung injury based on the criteria of Hill and US Surgeon General Expert Committee, all criteria were satisfactorily met. It was found that the form of lung injury reported in 2011 was caused by exposure to HDs. Above all, since no lung injury of unknown cause was reported before the sale of HD, and not a single case of lung injury has developed after the ban on HDs in November 2011, there was a strong support for the view that the lung injury of unknown cause was caused by exposure to HDs.

## Figures and Tables

**Table 1.** Epidemiological studies on the association between humidifier disinfectants and interstitial lung disease of unknown cause since 2011 performed in Korea

Author (publication year) [Ref]	Study subjects	Use of disinfectant vs. no use of disinfectant OR (95% CI)	Dose-response relationship OR, RR	Others
Case-control st udy				
Kim et al. (2014) [12]	No. of subjects (age range, yr) Patients: 18 (35.3, 44.0) Control: 121 (35.4, 42.9)	47.3 (6.1, 369.7)		Hospital control group Age, sex-matched Logistic regression model (no adjustment)
Yang et al. (2013) [13]	No. of subjects (age range, mo): Patients: 16 (18.25, 36.25; median, 26) Control: 47 (26.0, 29.5)	2.73 (1.41, 5.90)		Hospital control group Age, sex, first diagnosis-date matched Conditional logistic regression model
Park et al. (2016) [14]	No. of subjects (age range, yr) Patients: 16 (28.0, 49.0; median, 36.0) Control: 60 (27.0, 51.0; median, 35.0)	116.1 (6.5, 2,063.7)	According to 5-year cumulative exposure (L) OR: reference ((<0.5) -> 76.0 (0.5, 2.5) -> 272.9 (2.5+). According to daily exposure (mL/d) OR: reference (<10) -> 95.4 -> (10, 20) -> 133.5 (20+). According to 5-year exposure period (mo) OR: reference ((<5) -> 9.5 (5, 10) -> 52.9 (10+)	Community control group Age, sex, resident area, pregnancy history-matched Conditional logistic regression analysis
Park et al. (2015) [15]	No. of subjects (age range) Patients: 169 Control: 303 (≤6 yr and ≥35 yr, pregnant females included)		According to daily mean sleep time in a room with a humidifier containing disinfectants (hr/d) OR: reference (<10) ->1.7 (10.1, 11.0) -> 2.0 (11.1, 12.0) OR per the mean distance (m) between a humidifier and the patient’s bed (>1) -> 2.7 (0.5, 1.0) ->13.2 (<0.5). According to disinfectant concentration in the air (quartile, μg/m^3^) OR: reference (<317.1) -> 1.0 (317.2, 508.5) ->1.2 (508.6, 942.5) -> 2.6 (942.6, 4946.9)	Family control group No pairing Age, sex, factory within 1 km of resident area, number of chemical substances used at home Multivariate unconditional logistic regression analysis
Retrospective cohort study				
Paek et al. (2015) [16]	1,002 people, 273 families (death: 107) Age range: 0 to adult the exposed/the unexposed (549/408)	By age (vs. > 20 yr) 0-4: 3.84 (2.55, 5.79) 4-20: 1.89 (1.09, 3.27) Age-sex (vs. male adult) Female infants 17.14 (2.14, 137.59) Male infants 10.04 (1.23, 82.32) Female adults 6.02 (0.74, 49.10)	≥11 hr exposure/d (vs. <11 hr) 1.41 (0.90, 2.12) ≥7 d exposure/wk (vs. <7 d/wk) 4.07 (1.28, 12.91) ≥800 μg/m^3^ exposure (vs.<800 pg/m^3^) 1.61 (1.08, 2.40) Survival possibility according to exposure type: high-concentration constant exposure< low-concentration constant exposure < intermittent exposure	Survival analysis using Cox proportional hazards model

Ref, reference number; OR, odds ratio; CI, confidence interval; RR, risk ratio.

**Table 2.** Evaluation on the causality between humidifier disinfectants and lung injury based on Hill’s criteria

Criterion		Evidence	Met or not
Hill	US Surgeon General Expert Committee		
Strength of association	Strength of association	In a previous case-control epidemiological study, the OR of HD exposure (95% CI) was 47.3 (6.1, 369.7) in adults (hospital control group) [12], 116.1 (6.5, 2,063.7) (community control group) [14], and 2.73 (1.41, 5.90) in children [13], showing strong association	Met
Consistency	Replication of the findings	Association was found both in adults [12,14] and children [13], and in a case-control study, consistent results were found in different control groups (hospital [12,13], community [14], and family [15]); In addition, a significant association was reported not only in a case-control study, but also in a retrospective cohort study [16]	Met
Specificity	Specificity of the association	In an epidemiological study, lung disease of unknown cause could not be explained by other causes than HDs [12,13]; It was not consistent with clinical, radiological, and pathological findings of lung disease of other well-known causes such as viral, bacterial, or immunological causes [7,9]	Met
Temporality^1^	Temporal relationship	Lung injury of unknown cause had not been reported before HD was introduced to the market	Met
Biologic gradient	Dose-response relationship	As a result of a community-based case-control study, OR increased as the amount and period of HD use increased [14]; In a case-control study with a family control group, the increased exposure-OR association was shown according to sleep time, time to use humidifier per day, disinfectant concentration in the atmosphere, and the distance between a bed and humidifier in a room where humidifier containing disinfectant is turned on [15]; In a nationwide report of patients, lung injury or relevant mortality risk increased as the concentration was high in case of long and repetitive use of HD [16]	Met
Plausibility	Biological plausibility	As the size of aerosol containing HD sprayed through humidifier was <100 nm, it has been proven that only a small size can reach the peripheral bronchiole and get precipitated [1]; Lung injury was induced in an intra-tracheal drip animal study using a diluted concentration similar to the concentration of HDs that was on the market [1]	Met
Coherence to previous knowledge	Consistency of other knowledge	Previously known inhalation toxicity-induced lung lesions have characteristics such as lobular, diffuse infiltrative, and peri-bronchial infiltration, which were also shown in lung disease of unknown cause [7,9]; In toxicity evaluation reports published in other countries, there was no evaluation of inhalation toxicity as the major components of HDs had low volatility at room temperature. However, toxicity from oral exposure or dermatologic transmission was reported [1]	Met
Experiment		In a cellular toxicity experiment exposing normal pulmonary cells to the major components of HD, dose-dependent toxicity was expressed, and dose-dependently reactive oxygen was developed as a result of evaluating reactive oxygen production [1]; In an inhalation animal study using diluted concentration that was present on the market, histopathological findings similar to those found in patients with lung disease of unknown cause were observed [17]	Met
Analogy		Ardystil syndrome, an interstitial lung disease developed in workers using spray paint in a western country in the 1990s, is similar to the case of lung disease due to HDs; A component of paint, acramin, has a very similar chemical structure to that of polyhexamethylene guanidine , which is found in HDs [18]	Met
	Consideration of alternative explanations	In an epidemiological study, the degree of the association between fungi and lung injury was substantially small compared to the association between HDs and lung injury; In other epidemiological studies [12,13], as there was no association between fungi and lung injury, presence of fungi can be interpreted as resulting from the use of the humidifiers; In case of hypersensitivity pneumonitis (humidifier fever) caused by toxins (endotoxin) from bacteria colonizing in humidifier [19] and white-dust related fever due to inhalation of metal deposits such as calcium and magnesium included in the water of humidifiers [20], there is distinctive difference in clinical, radiological and pathological findings compared to HD-induced lung injury	Met
	Cessation of exposure^1^	There is no new incidence since HDs have been withdrawn from the market in November 2011; Despite its withdrawal, disease progress was irreversible in patients who had developed the disease before	Met
			

OR, odds ratio; CI, confidence interval; HD, humidifier disinfectant.

1 Hill’s criteria do not have exposure cessation items, the fact that there is no disease incidence after exposure cessation can be interpreted as temporal relationship.

## References

[1] Lung Injury Investigation Committee, Korea Centers for Disease Control and Prevention (2014). Report of the incidence of humidifier disinfectant-associated damages.

[2] Höfler M (2005). Causal inference based on counterfactuals. BMC Med Res Methodol.

[3] Szklo M, Nieto J (2014). Epidemiology: beyond the basics.

[4] Hill AB (1965). The environment and disease: association or causation?. Proc R Soc Med.

[5] Gordis L (2014). Epidemiology.

[6] Koo HJ, Do KH, Chae EJ, Kim HJ, Song JS, Jang SJ (2016). Humidifier disinfectant-associated lung injury in adults: prognostic factors in predicting short-term outcome. Eur Radiol.

[7] Lee E, Seo JH, Kim HY, Yu J, Jhang WK, Park SJ (2013). Toxic inhalational injury-associated interstitial lung disease in children. J Korean Med Sci.

[8] Kim KW, Ahn K, Yang HJ, Lee S, Park JD, Kim WK (2014). Humidifier disinfectant-associated children’s interstitial lung disease. Am J Respir Crit Care Med.

[9] Hong SB, Kim HJ, Huh JW, Do KH, Jang SJ, Song JS (2014). A cluster of lung injury associated with home humidifier use: clinical, radiological and pathological description of a new syndrome. Thorax.

[10] Yoon HM, Lee E, Lee JS, Do KH, Jung AY, Yoon CH (2016). Humidifier disinfectant-associated children’s interstitial lung disease: computed tomographic features, histopathologic correlation and comparison between survivors and non-survivors. Eur Radiol.

[11] Kim YH, Kim KW, Lee KE, Lee MJ, Kim SK, Kim SH (2016). Transforming growth factor-beta 1 in humidifier disinfectant-associated children’s interstitial lung disease. Pediatr Pulmonol.

[12] Kim HJ, Lee MS, Hong SB, Huh JW, Do KH, Jang SJ (2014). A cluster of lung injury cases associated with home humidifier use: an epidemiological investigation. Thorax.

[13] Yang HJ, Kim HJ, Yu J, Lee E, Jung YH, Kim HY (2013). Inhalation toxicity of humidifier disinfectants as a risk factor of children’s interstitial lung disease in Korea: a case-control study. PLoS One.

[14] Park JH, Kim HJ, Kwon GY, Gwack J, Park YJ, Youn SK (2016). Humidifier disinfectants are a cause of lung injury among adults in South Korea: a community-based case-control study. PLoS One.

[15] Park DU, Choi YY, Ahn JJ, Lim HK, Kim SK, Roh HS (2015). Relationship between exposure to household humidifier disinfectants and risk of lung injury: a family-based study. PLoS One.

[16] Paek D, Koh Y, Park DU, Cheong HK, Do KH, Lim CM (2015). Nationwide study of humidifier disinfectant lung injury in South Korea, 1994-2011. Incidence and dose-response relationships. Ann Am Thorac Soc.

[17] Park S, Lee K, Lee EJ, Lee SY, In KH, Kim HK (2014). Humidifier disinfectant-associated interstitial lung disease in an animal model induced by polyhexamethylene guanidine aerosol. Am J Respir Crit Care Med.

[18] Nemery B, Hoet PH (2015). Humidifier disinfectant-associated interstitial lung disease and the Ardystil syndrome. Am J Respir Crit Care Med.

[19] (1978). Humidifier fever: a disease to look out for. Br Med J.

[20] Daftary AS, Deterding RR (2011). Inhalational lung injury associated with humidifier “white dust”. Pediatrics.

[21] Kim JY, Kim HH, Cho KH (2013). Acute cardiovascular toxicity of sterilizers, PHMG, and PGH: severe inflammation in human cells and heart failure in zebrafish. Cardiovasc Toxicol.

