# Supplementary material for: Evaluation report on the causal association between humidifier disinfectants and lung injury
Source: Epidemiol Health. 2016 Aug 18;38:e2016037. doi: 10.4178/epih.e2016037 (PMC5081304; doi:10.4178/epih.e2016037)
Supplement: Supplementary file 1 [file epih-38-e2016037-supplementary.pdf]

가습기살균제와 원인미상 중증폐질환의 관련성에 대한 인과성 평가

Evaluation Report on the Causal Association between Humidifier Disinfectants and Unknown Cause Severe Lung Diseases

하미나[1,8], 이순영[2,8], 황승식[3,8], 박혜숙[4,8], 신승수[5,8], 정해관[6], 최보율[7]

[1]단국대학교의과대학예방의학교실, [2]아주대학교 의과대학 예방의학교실, [3]인하대학교 의과대학 사회의학교실, [4]이화여자대학교 의과대학 예방의학교실,[5]아주대학교 의과대학 내과학교실, [6]성균관대학교 의과대학 사회의학교실, [7]한양대학교 의과대학 예방의학교실, [8]한국역학회 학술위원회

교신저자: 이순영, 아주대학교 의과대학 예방의학교실, solee5301@gmail.com

Running title

Humidifier Disinfectants and Unknown Cause Severe Lung Diseases

가습기살균제와 원인미상 중증폐질환의 연관성에 대한 인과성 평가

Evaluation Report on the Causal Association between Humidifier Disinfectants and Unknown Cause Severe Lung Diseases

Abstract

As of November 2011, Korean government recalled and banned the humidifier disinfectants from the markets since 4 case-control studies and 1 retrospective epidemiological studies were published to prove the association between humidifier disinfectants and severe interstitial lung disease with unknown cause. After a careful examination on the association between the humidifier disinfectants and sever lung diseases based on the Hill's criteria, we found that all the evidences on the causality fulfilled the 9 criteria (strength of association, consistency, specificity, temporality, biologic gradient, plausibility, coherence, experiment, and analogy), which proved the unknown cause severe lung diseases reported in 2011 might be caused by the disinfectants. In specific, there was no single reported case of unknown cause severe lung diseases since the ban of selling the humidifier disinfectants in November, 2011 which also strongly proved the overarching temporal association between the diseases and the disinfectants, the most necessary one of Hill's criteria. Although the number of epidemiological studies regarding the humidifier disinfectants case occurred only in Korea were few, those studies contributed to prove the strong association between the disinfectants and the severe lung diseases based on the scientific evidences.

Key words: causality, Humidifiers, interstitial lung disease, case-control studies

1. 서론

가습기살균제 사고는 세계적으로도 유례없는 생활용품에 의한 대규모의 환경참사이다. 2011년 4월에 서울시내 한 대학병원 감염관리실의 신고를 받은 질병관리본부의 역학조사를 시작으로, 가습기살균제의 인체 건강손상, 특히 원인미상의 중증폐질환과의 연관성에 대한 연구들이 수행되었고 이 연구결과들로부터 사건원인에 대한 근거가 도출되기 시작하였다. 2011년 11월에 정부는 이 근거에 기반하여 추가적인 피해발생을 막기 위해 가습기살균제를 시중에서 회수하고 유통되지 못하도록 하는 조치를 취하였고, 이후 이와 유사한 중증폐질환자가 한 명도 발생하지 않았다 [1].

본 고에서는 가습기살균제가 원인미상의 중증호흡기폐질환의 원인이라는 점을 과학적 연구결과에 근거하여 고찰하였다.

2. 연관성과 인과성

인구집단에서 어떤 노출 요인이 특정 질병의 원인임이 증명되기 위해서 종종 도입되는 개념이 counterfactual concept 이다. 이것은 어떤 요인이 특정 질병의 원인인가를 확인하기 위해서는, 요인에 노출되었을 때(fact)의 질병발생률을 이와 동일한 대상(개인 혹은 인구집단)이 동일한 환경에서 해당 요인에 노출되지 않았을 때(counter-fact)의 질병발생률과 비교함으로써, 인과성 여부를 결정할 수 있다고 판단한다 [2]. 그러나 실제 현실에서는 fact 는 관찰할 수는 있어도 동일한 상황에서의 counter-fact 를 관찰하는 것은 불가능하다. 대신에 완전 동일하지는 않지만 유사한 특성을 가진 대상에서, 유사한 환경하에 fact 와 다른 노출조건에서의 질병발생률을 관찰하여 비교하는 방법을 취하게 된다. 여기에서 산출되는 것은 노출요인과 질병간의 연관성(association)이다. 따라서 이것은 fact 와 counter-fact 에서의 질병발생률을 비교하여 얻을 수 있는 인과성(causation)과는 다르다. 즉, 유사하지만 동일하지는 않은 대상과 환경이라는 조건 때문에, 이들간 차이 나는 변수들에 의해 교란효과 및 매개효과 등이 발생하게 된다.

따라서 역학적 관찰의 결과 얻은 연관성을 인과성으로 해석할 수 있으려면, 여러 가지 측면에서 논리적 추론이 필요하다. 즉, 역학적 연구에서 관찰된 연관성이 각종 비뚤림과 우연에 의한(통계적 유의성) 것이 아님을 확인한 후, 인과성 판단의 기준을 충족하는지 여부를 판단하여야 한다 [3].

최근까지도 특히 만성질환 관련 인과성을 설명하기 위한 모델들이 제시되고 있지만, 본고에서는 역학자와 건강정책전문가에게 여전히 인과성 추정에 실용적인 기준으로 널리 통용되고 있는 [3] Bradford Hill의 인과성 판단의 기준에 입각하여 평가해 보고자 한다 [4].

3. 가습기살균제와 중증폐질환의 연관성을 보고한 역학적 연구

원인미상 중증폐질환의 임상적 영상의학적 병리학적 양상과 예후에 대한 사례군 연구는 2016년 현재까지 총 6편이 발표되었고 [5-10], 가습기살균제와의 연관성에 관한 추정치가 산출된 역학연구는 현재까지 총 5편이 발표되었다 [11-15] (Table 1).

--- Table 1 placed here-----

환자-대조군 연구는 총 4편으로서 2011년 한 대학병원에서 원인미상 중증폐질환이 질병관리본부에 신고된 후 즉각적으로 그리고 최초로 시행된 역학조사 [11], 어린이를 대상으로 한 3배수로 성, 연령, 진단일을 짝지은 환자-병원대조군 연구 [12], 지역사회 대조군을 성, 연령, 거주지역과 출산경험유무를 짝지은 환자-대조군 연구 [13], 그리고 가족 내 대조군과 환자를 비교하여, 환자가 다른 가족에 비하여 가습기에 더 가깝게, 더 많은 시간 동안 노출되었음을 보고한 연구들이다 [14]. 이 연구들에서 교란변수로 인한 비뚤림을 배제하기 위해 주요 교란변수에 대해 짝을 짓거나, 분석과정에서 통계학적 보정을 하였다. 특히 최초로 수행된 역학조사 [11]는 환자나 조사자가 가습기살균제가 의심요인 중 하나라는 사실을 인지하지 못한 시점이어서 회상비뚤림의 여지가 최소화되었다고 판단할 수 있다. 가족 대조군을 사용한 연구를 제외한 3편의 환자-대조군 연구는 환자수가 16명 혹은 18명으로 매우 적은 숫자이나 환자군의 90-100%에서 가습기살균제를 사용하였던 것으로 보고되어, 통계적인 우연의 여지도 배제할 수 있다고 판단된다. 가습기살균제 피해자 접수결과 모집된 전국적 환자를 대상으로 한 후향적 코호트 연구 [15]에서는 여성, 어린이가 더 큰 위험을, 또 노출용량에 비례하여 위험도가 상승하였음을 밝혔다.

4. Hill의 인과성기준에 따른 인과성의 근거평가

수행된 역학연구와 임상적, 독성학적 및 실험적 연구 결과에 기반하여, 가습기 살균제와 중증 폐질환간의 인과성을 Hill의 9가지 기준에 적용하여 논의하고자 한다(표 2).

--- Table 2 placed here -----

첫째, 연관성의 강도이다. 위에서 언급한 역학연구 결과에 따르면 가습기살균제와 원인미상 중증폐질환 사이에 적게는 2.73배, 많게는 116배에 이르러 통계학적으로 유의한 강한 연관성이 인정된다 [11-13].

둘째, 일관성이다. 각각의 역학연구는 다른 인구집단(성인 [11-13] vs. 소아 [12]), 다른 연구설계 (환자-대조군 [11-14]vs. 후향적 코호트 [15]) 또는 다른 대조군(병원대조군 [11, 12], 지역사회대조군 [13], 가족대조군 [14])을 가진 연구였음에도 불구하고 모두 일관된 연관성을 보였다.

셋째, 특이성이다. 원인미상 중증폐질환은 다른 바이러스나 세균 등의 감염성 폐질환이나, 면역성 폐질환 등 기존에 알려진 경우로 모두 설명되지 않는 임상적 및 병리적 소견으로 [6-8], 역학적 연구에서 가습기살균제 이외의 다른 환경적 요인으로도 설명되지 못하였다 [11, 12]. 이러한 면에서 가습기살균제 노출과 중증폐질환 발생간에 특이적 관계가 있이 인정된다.

넷째, 시간적 선후관계이다. 원인미상 중증폐질환이 가습기살균제가 시판되기 이전시기에는 보고된 바가 없었고, 2011년 11월 전면 판매 중지조치가 내려진 이후에 추가 발생 사례가 전혀 없었음을 고려할 때, 시간적 선후관계의 조건이 충족된다.

다섯째, 용량-반응관계이다. 3편의 역학연구에서 가습기살균제에 더 많이, 더 오래, 더 가깝게 노출될수록 발생 및 사망위험이 더 큰 일관된 결과를 보이고 있다 [13-15].

여섯째, 개연성이다. 가습기를 통해 분무된 에어로졸의 크기가 30-50nm 가 가장 많았고 이 크기의 입자는 세기관기까지 도달하여 침착될 수 있음이 밝혀져 [1], 가습기살균제의 화학물질이 에어로졸을 통해 세기관지에 도착할 수 있음을 시사하였다. 또한, 실제 사용농도로 희석된 가습기살균제액을 동물의 기관지에 점적한 결과 폐손상이 유발되는 것이 확인되어, 실제 사용농도의 묽은 가습기살균제가 독성을 유발할 수 있음을 시사하였다. 이러한 결과들은 가습기살균제가 희석된 가습기의 분무가 흡입을 통해 소기관지까지 도달, 폐 손상을 유발할 수 있는 개연성을 보여준

다.

일곱째, 기존지식과의 일치성이다. 기존에 알려져 있던 흡입독성에 의한 폐병변이 주로 소엽 중심성, 미만성 침윤 및 기관지주위 침윤 등의 특성을 보이는데, 원인미상 중증폐질환의 경우도 이와 일치하는 폐병변을 보였다 [6, 8]. 호주나 미국에서는 살균제액을 물에 타서 가습기살균제 용도로 사용될 것이라는 것을 염두에 두지 않았기 때문에, 독성평가자료에서 흡입독성에 대한 자료는 없었다. 그러나 가습기살균제의 주요 성분들이 상온에서 휘발성이 낮다는 사유로 흡입독성에 대한 평가가 없었을 뿐 경구 혹은 피부접촉을 통한 다양한 독성은 이미 보고되고 있었다 [1]. 이러한 사실들은 가습기살균제와 원인미상 중증폐질환 간의 연관성이 기존지식과 일치한다는 점을 보여준다.

여덟째, 실험적 근거이다. 가습기살균제의 주요 성분에 대한 정상폐세포에 노출시킨 세포독성 실험에서 용량-의존적인 독성이 발견되었고, 활성산소 생성평가 결과 또한 용량-의존적으로 발생함을 확인하였다 [1]. 실제 사용농도로 희석한 가습기살균제의 흡입 동물실험에서 원인미상 중증폐질환 환자의 폐 병리소견과 동일한 조직병리 소견이 재현됨을 확인하였다 [16].

마지막으로, 유사성이다 1990년대 서구에서 스프레이 페인트 사용 근로자에서 발생한 간질성 폐질환인 Ardystil 증후군은 가습기살균제로 인한 폐질환과 유사한 사례라고 할 수 있다 [17]. 이것은 페인트에 들어있던 acramin성분이 가습기살균제의 PHMG 와 매우 유사한 화학적 구조를 가진다는 점에서 유사한 사례라고 하겠다.

4. 인과성 평가 종합 및 역학적 인과성 고찰에서 고려해야 할 사항

종합하면 매우 이례적이게도 가습기살균제와 원인미상 중증폐질환의 연관성은 Hill의 인과성 기준 9항목을 모두 충족하였다. 따라서 가습기살균제는 중증폐질환의 원인이라는 점에 대하여 매우 강력한 과학적 근거가 존재한다고 할 수 있다. 그러나, 가습기살균제의 흡입독성이, 중증폐질환에 국한되어 나타난다고 할 수는 없다. 이미 출판된 기존 보고에서 지적되고 있듯이, 중증폐질환은 노출수준이 매우 높은 고위험군에서 발생하는 질병으로 생각되며, 이보다 낮은 수준의 노출자에서는 보다 경미한 질환이 발생했을 가능성이 있다. 또 폐가 아닌 다른 장기에서의 손상 또한 가능할 것임이 의심되고 있고 실제로 신고된 피해자들에서 비염이나 천식 등의 증상을 호소하는

경우가 많고, 폐가 아닌 다른 장기의 질환과 증상 또한 보고하고 있다 [15]. 뿐만 아니라, 어류독성실험에서 간손상 및 동맥경화를 포함한 심혈관계 이상소견이 관찰된 바 있어 [18] 이러한 의심을 뒷받침하는 근거가 되고 있다. 이것은 가습기살균제가 중증폐질환 발생하고만 관련이 있다고 할 수 없으며, 즉, 가습기살균제와 중증폐질환의 특이성을 부정하는 소견일 수 있다. 그러나 폐암의 강력한 원인이지만 다양한 질환 발생에 기여하는 흡연의 예처럼 다량의 가습기살균제의 노출은 중증 폐질환 발생과 특이성이 있으나, 가습기살균제 노출수준이 상대적으로 낮은 경우에서는 기존 폐질환의 악화, 천식이나 비염 등의 비특이적인 증상으로 발현될 수 있는 것으로 판단할 수 있다. 실제로 특이성은 인과관계 고찰에서 필수적인 조건은 아니다 [3].

대부분의 환경성 질환은 단일 요인에 의하여 발생되지 않는다. 필수(necessary cause) 혹은 충분(sufficient cause) 역할을 하는 여러 가지 요인 [3]들이 동시에 복합적으로 작용한다고 보는 것이 더 진실에 가깝다. 또한 미시적 수준의 생화학적 반응에서부터 개인적 수준의 행동적 요인, 나아가 물리적 화학적 사회적 심리적 환경이라는 거시적 수준의 요인까지 복합적 다차원 수준에서의 상호작용에 의해 영향을 받으며 발생된다 [3]. 원인미상 중증폐질환 연구들에서 가습기살균제 이외의 다른 유력한 위험요인은 존재하지 않았으나, 첫번째 역학연구에서 곰팡이 노출이 여러 변수를 보정하고도 중증폐질환과 유의한 연관성을 보였다 [11]. 물론 가습기살균제보다는 현저히 작은 크기의 연관성이고, 일부 대조군별 분석에서는 유의성이 없어 결과의 일관성이 없었으며, 검체분석에서 곰팡이가 검출되지 않은 점을 고려해 볼 때 곰팡이가 중증폐질환의 직접적인 원인일 가능성은 매우 낮은 것으로 판단되었다.

따라서 이번 가습기살균제 사건의 경우, 곰팡이 자체가 질병의 원인으로 작용하였다기 보다는 오래된 가습기 사용 자체가 곰팡이 증식을 유발한 것으로 판단하는 것이 타당하다. 그러나 중증폐질환 발생에 가습기살균제가 다른 요인들과의 상호작용, 개인적인 감수성과의 상호작용, 문제극복능력(coping capacity)을 결정하는 경제적 사회적 역량과의 상호작용 등이 모두 관여하였을 것을 배제하기는 어렵다. 즉, 가습기살균제는 중증폐질환 발생의 필수 요인으로 직접적 원인이 명확하나, 다른 가능한 요인은 질병발생의 구성원인으로 서 중증폐질환 발생과정에 공존할 수 있음을 의미한다. 어떤 요인과 특정 질병발생간의 인과성을 밝히는 것은 질병을 예방하고 치료하는데 가장 근본적이지만, 대부분의 질환 발생에 비특이성, 다요인의 공존가능성, 다요인간의 상호작용

의 가능성 등을 고려하여 인과성을 결론내기는 쉽지 않다. 2011년 가습기살균제 사건은 국내에서 유일하게 발생된 사건으로 역학연구의 수가 많지는 않았지만, 과학적이고 신속하게 질병의 원인을 규명하는데 큰 기여를 했다는 의미가 크다.

결론적으로 가습기살균제와 중증폐질환간의 인과성을 Hill의 기준으로 검토한 결과 9개 조건을 모두 충족하였고, 2011년 발생한 원인미상 중증폐질환의 원인이 가습기살균제 노출로 인한 것임을 알 수 있었다. 특히 2011년 11월 가습기살균제 판매중지 이후 원인미상 중증폐질환이 한 건도 발생하지 않은 것은 Hill의 기준 중 필수요소인 시간적 선후관계 [3]를 강력하게 지지하는 가장 중요한 증거라고 할 수 있다.

참고문헌

1.보건복지부 질병관리본부 폐손상조사위원회. 가습기살균제 건강피해 사건 백서. 서울. 도서출판 한림원. 2014

2.Höfler M. Causal inference based on counterfactuals. BMC Med Res Methodol 2005;5:1.

3.Szklo M, Nieto J. Epidemiology: Jones & Bartlett Publishers; 2012

4.Hill AB. The environment and disease: association or causation? Proc R Soc Med 1965;58:295.

5.Koo HJ, Do K-H, Chae EJ, Kim HJ, Song JS, Jang SJ, et al. Humidifier disinfectant-associated lung injury in adults: Prognostic factors in predicting short-term outcome. Eur Radiol 2016:1-9.

6.Lee E, Seo J-H, Kim HY, Yu J, Jhang W-K, Park S-J, et al. Toxic inhalational injury-associated interstitial lung disease in children. J Korean Med Sci 2013;28:915-923.

7.Kim KW, Ahn K, Yang HJ, Lee S, Park JD, Kim WK, et al. Humidifier disinfectant–associated children's interstitial lung disease. Am J Respir Crit Care Med 2014;189:48-56.

8.Hong S, Kim H, Huh J, Do K, Jang S, Song J, et al. Korean Unknown Severe Respiratory Failure Collaborative Korean Study Group of Respiratory Failure A cluster of lung injury associated with home humidifier use: clinical, radiological and pathological description of a new syndrome. Thorax 2014;69:694-702.

9.Yoon HM, Lee E, Lee JS, Do K-H, Jung AY, Yoon CH, et al. Humidifier disinfectant-associated children's interstitial lung disease: Computed tomographic features, histopathologic correlation and comparison between survivors and non-survivors. Eur Radiol 2016;26:235-243.

10.Kim YH, Kim KW, Lee KE, Lee MJ, Kim SK, Kim SH, et al. Transforming growth factor-beta 1 in humidifier disinfectant-associated children's interstitial lung disease. Pediatr Pulmonol 2016;51:173-182.

11.Kim HJ, Lee M-S, Hong S-B, Huh JW, Do K-H, Jang SJ, et al. A cluster of lung injury cases associated with home humidifier use: an epidemiological investigation. Thorax 2014:thoraxjnl-2013-204132.

12.Yang H-J, Kim H-J, Yu J, Lee E, Jung Y-H, Kim H-Y, et al. Inhalation toxicity of humidifier disinfectants as a risk factor of children's interstitial lung disease in Korea: a case-control study. PLoS One 2013;8:e64430.

13.Park J-H, Kim HJ, Kwon G-Y, Gwack J, Park Y-J, Youn S-K, et al. Humidifier Disinfectants Are a Cause of Lung Injury among Adults in South Korea: A Community-Based Case-Control Study. PLoS One 2016;11:e0151849.

14.Park D-U, Choi Y-Y, Ahn J-J, Lim H-K, Kim S-K, Roh H-S, et al. Relationship between Exposure to Household Humidifier Disinfectants and Risk of Lung Injury: A Family-Based Study. PLoS One 2015;10:e0124610.

15.Paek D, Koh Y, Park D-U, Cheong H-K, Do K-H, Lim C-M, et al. Nationwide Study of Humidifier Disinfectant Lung Injury in South Korea, 1994–2011. Incidence and Dose–Response Relationships. Ann Am Thorac Soc 2015;12:1813-1821.

16.Park S, Lee K, Lee EJ, Lee SY, In KH, Kim H-K, et al. Humidifier Disinfectant–associated Interstitial Lung Disease in an Animal Model Induced by Polyhexamethylene Guanidine Aerosol. Am J Respir

Crit Care Med 2014;190:706-708.
17.Nemery B, Hoet PH. Humidifier Disinfectant–associated Interstitial Lung Disease and the Ardystil Syndrome. Am J Respir Crit Care Med 2015;191:116-117.
18.Kim J-Y, Kim HH, Cho K-H. Acute cardiovascular toxicity of sterilizers, PHMG, and PGH: severe inflammation in human cells and heart failure in zebrafish. Cardiovasc Toxicol 2013;13:148-160.

국문초록
2011년 4월 이후 국내에서는 가습기살균제와 원인미상 증증폐질환의 연관성을 밝히기 위하여 4개의 환자-대조군 연구와 1개의 후향적 역학연구가 수행되었으며, 이를 근거로 한국전부는 같은 해 11월 가습기살균제를 시중에서 회수하고 유통되지 못하도록 하는 조치를 취하였다. 본 원고에서는 Hill의 기준에 근거하여 가습기살균제와 원인미상 중증폐질환간의 인과성을 밝히기 위하여 으로 검토한 결과 9개 조건(연관성의 강도, 일관성, 특이성, 시간적 선후관계, 생물학적 용량-반응 관계, 개연성, 기존 지식과의 일치성, 실험 및 유사성) 을 모두 충족함을 확인하므로 써, 2011년 발생한 원인미상 중증폐질환의 원인이 가습기살균제 노출로 인한 것임을 알 수 있었다. 특히 2011년 11월 가습기살균제 판매중지 이후 원인미상 중증폐질환이 한 건도 발생하지 않은 것은 Hill의 기준 중 필수요소인 시간적 선후관계[3]를 강력하게 지지하는 가장 중요한 증거라고 할 수 있다. 이번 가습기살균제 사건은 국내에서 유일하게 발생된 사건으로 역학연구의 수가 많지는 않았지만, 과학적이고 신속하게 질병의 원인을 규명하는데 큰 기여를 했다는 의미가 크다.
